# Shaking Up the Synthesis of Organophosphorus Compounds via Ortho-phosphite

**DOI:** 10.1021/acscentsci.5c02384

**Published:** 2026-01-06

**Authors:** Alexandros Paparakis, Andrew R. Jupp

**Affiliations:** School of Chemistry, 1724 University of Birmingham, Edgbaston, Birmingham B15 2TT, U.K.

## Abstract

Mechanochemistry enables the preparation of the fundamental ortho-phosphite [PO_3_]^3−^ anion from condensed phosphates.

## Graphical abstract

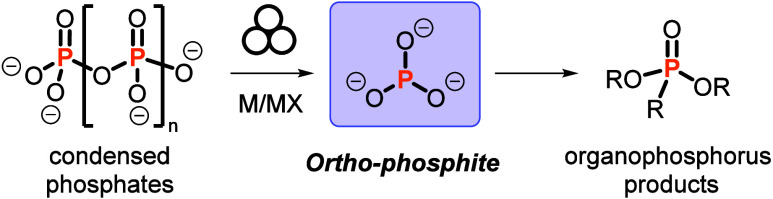

Mechanochemistry has been carried out in various forms for millennia,^1^ and in more recent years it has emerged as a viable synthetic methodology to mitigate solvent use in a variety of chemical transformations.^2^ Furthermore, it has enabled the preparation of novel molecules and materials that are not possible under conventional solution-phase conditions. The efficiency and scalability of mechanochemical techniques means they are not just useful for academic curiosities, but can be well-suited for developing sustainable industrial processes of the future.^3^

Phosphorus is an essential element for all known forms of life; it forms the structural backbone of nucleic acids (DNA and RNA), acts as a key component of energy carriers such as ATP, participates in phosphorylation reactions essential for enzyme regulation and signal transduction, and forms phospholipid bilayers that contribute to the stability and function of cells.^4^ Industrially, phosphorus is derived from phosphate rock, most commonly fluoroapatite. Typically, phosphate rock is converted to phosphoric acid via acidulation and used to make fertilizers, whereas (organo)phosphorus fine chemicals are typically synthesized via the reduction of phosphate rock to white phosphorus (P_4_) and subsequent functionalization. Pertinent work from Cummins^5^ and Weigand^6^ has started revealing pathways for the conversion of phosphate directly into valorized organophosphorus products, circumventing the need for the high-energy reduction reaction to white phosphorus.^7^

In this issue of *ACS Central Science*, Cummins and co-workers report the first synthesis of ortho-phosphite [PO_3_]^3–^ by leveraging the mechanochemical reduction of condensed phosphates.^8^ [PO_3_]^3–^ is a potential source of phosphorus to create new value chains for P-containing chemicals as an alternative to white phosphorus. Despite being used as a model Lewis structure in the classroom, [PO_3_]^3–^ is elusive and this report is the first synthesis of this seemingly simple anion.

Despite being used as a model Lewis structure in the classroom, [PO_3_]^3–^ is elusive and this report is the first synthesis of this seemingly simple anion.

However, heavier congeners of ortho-phosphite do exist, as there are known natural examples and synthetic examples of other [EO_3_]^3–^ anions (E = As, Sb, Bi) like Reinerite (Zn_3_(AsO_3_)_2_), K_3_SbO_3_, or Na_3_BiO_3_.^9,10^ Other fundamental phosphorus oxoanions like [PO_4_]^3–^, [HPO_3_]^2–^, and [PO_3_]^−^ compounds are also well established, as either components of naturally occurring minerals or synthetically; this led the researchers to dub [PO_3_]^3–^ as the “missing oxoanion” of phosphorus.

To find this missing oxoanion, Cummins and co-workers reduced condensed phosphates to ortho-phosphite via ball milling in the presence of highly active reducing agents: alkali-metal dispersions on MX salts like K/KI and Na/NaCl (Figure 1). Building on recent work from Pearce and co-workers,^11^ they showed that K/KI and Na/NaCl reducing agents could also be readily prepared using mechanochemical methods, thus demonstrating the power of mechanochemistry for the entire process. A range of condensed phosphates were amenable to the methodology, exemplifying the broad applicability of this reduction chemistry. The ortho-phosphite in the mixture was characterized by solid-state ^31^P NMR spectroscopy and Raman spectroscopy, and by comparing the values with computed values for the vibrational bands. Further verification of the ortho-phosphite was elegantly demonstrated by protonating the mixture with H_2_O (or D_2_O) and observing the resulting known phosphite anion [HPO_3_]^2–^ (or [DPO_3_]^2–^).

**Figure 1:**
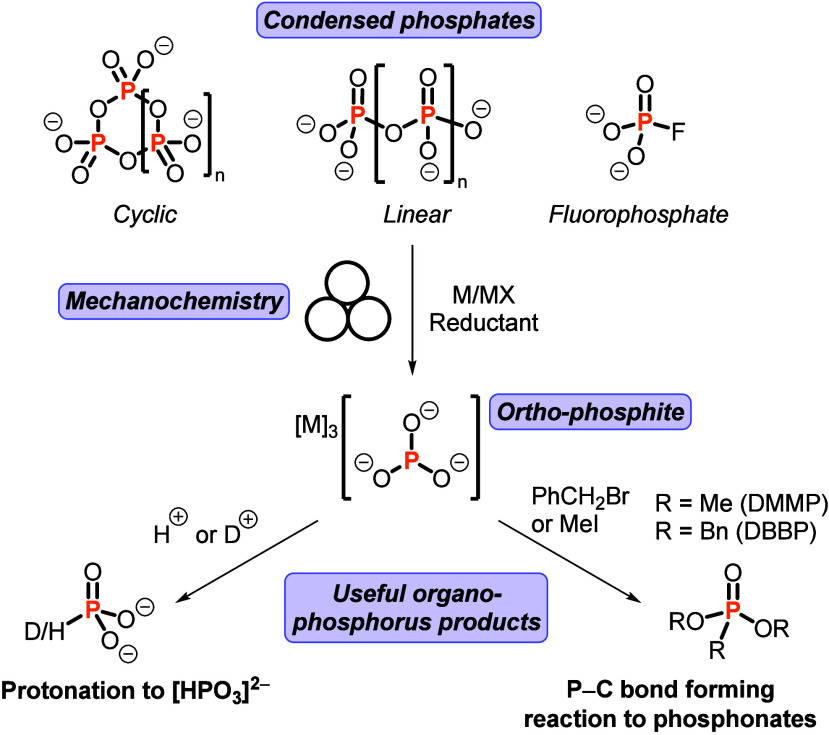
Mechanochemical reduction of condensed phosphates allows for the synthesis of the fundamental ortho-phosphite anion, which can be valorized into phosphorus fine chemicals.

After successfully demonstrating reduction from condensed phosphates to [PO_3_]^3–^, they demonstrated the utility of the new phosphorus source by valorizing alkali metal ortho-phosphite salts in a variety of ways. Due to the difficulties in purifying the ortho-phosphite, the crude mixture was treated with reagents for the functionalization reactions. The aforementioned phosphite anion [HPO_3_]^2–^ is a fungicide and fertilizer, and is typically obtained from P_4_ (via PCl_3_). Other important P­(III) chemicals were synthesized from the crude mixture, including P­(OSiMe_3_)_3_, which is a common ligand and battery electrolyte, and can itself be readily valorized to phosphonates via the Michaelis–Arbuzov reaction. P­(V) organophosphorus compounds were also readily synthesized from the mixture containing [PO_3_]^3–^; the addition of benzyl bromide or methyl iodide resulted in new P–C bond formations, and the preparation of dibenzyl phosphonate (DBBP) or dimethyl phosphonate (DMMP), respectively (Figure 1). These proof-of-concept examples for the formation of useful P­(III) and P­(V) compounds via ortho-phosphite represent a new way to access these compounds and open many future avenues of research. It will be interesting to see how the authors, or others, tackle the following questions:

- Can the ortho-phosphite be isolated cleanly? Both the reduction of condensed phosphates and the deprotonation of [HPO_3_]^2–^ led to mixtures with other phosphorus-containing products.
- Can the ortho-phosphite be crystallographically characterized?
- Can the yields for the functionalization reactions be increased? For example, the DMMP and DBBP (Figure 1) are synthesized in 28% and 32% yields, respectively, with respect to ortho-phosphite in the mixture, but this drops to 9% and 10%, respectively, when you consider the amount of phosphorus in the condensed phosphate that is used initially. This will improve naturally if the ortho-phosphite can be isolated cleanly in the first place, but for now this is a challenge that needs to be overcome to make this an attractive route for the synthesis of organophosphorus compounds.
- What other useful or novel P­(III) and P­(V) compounds can be accessed via this route, and at what scale?

These proof-of-concept examples for the formation of useful P­(III) and P­(V) compounds via ortho-phosphite represent a new way to access these compounds and open many future avenues of research.

In summary, Cummins and co-workers have identified and synthesized a fundamental phosphorus oxoanion species via the mechanochemical reduction of different condensed phosphate precursors. The ortho-phosphite could then be transformed into a range of phosphorus products that are typically accessed via P_4_. There are questions that arise regarding the purity of the ortho-phosphite and the scope, yield, and scalability of the downstream phosphorus products, but this work is the starting point for a new way of converting phosphates into important phosphorus-based fine chemicals in a more efficient manner.
